# Detailed statistical analysis plan for the neurological complications in endoscopic versus open radial artery harvest (NEO) randomised clinical trial

**DOI:** 10.1186/s13063-022-06869-7

**Published:** 2022-12-09

**Authors:** Christian L. Carranza, Martin Ballegaard, Mads U. Werner, Philip Hasbak, Andreas Kjaer, Klaus Kofoed, Markus Harboe Olsen, Christian Gluud, Janus Christian Jakobsen

**Affiliations:** 1 Department of Cardio-Thoracic Surgery, The Heart Centre, Copenhagen University Hospital − Rigshospitalet, Copenhagen, Denmark; 2 Department of Neurology, Zealand University Hospital, Roskilde, Denmark; 3 Multidisciplinary Pain Centre, Department of Anesthesia, Pain and Respiratory Support, Neuroscience Center, Copenhagen University Hospital − Rigshospitalet, Copenhagen, Denmark; 4 Department of Clinical Physiology, Nuclear Medicine and PET and Cluster for Molecular Imaging, Copenhagen University Hospital – Rigshospitalet, Copenhagen, Denmark; 5 Department of Biomedical Sciences, University of Copenhagen, Copenhagen, Denmark; 6 Department of Cardiology and Radiology, Copenhagen University Hospital − Rigshospitalet, Copenhagen, Denmark; 7 Centre for Clinical Intervention Research, Copenhagen Trial Unit, The Capital Region, Copenhagen University Hospital − Rigshospitalet, Copenhagen, Denmark; 8 Department of Neuroanaesthesiology, The Neuroscience Centre, Copenhagen University Hospital − Rigshospitalet, Copenhagen, Denmark; 9 Department of Regional Health Research, The Faculty of Health Sciences, University of Southern Denmark, Odense, Denmark

## Abstract

**Introduction:**

Coronary artery bypass grafting can be conducted using the radial artery as a bypass graft. However, it remains unclear which harvesting method is superior, i.e. endoscopic or open radial artery, and which site for proximal anastomosis of the radial artery has the greatest benefits?

**Methods:**

The NEO Trial is a single site randomised clinical trial with a 2 × 2 factorial design. The first comparison assesses endoscopic versus open radial artery harvest with a primary outcome of hand function and secondary outcomes of neurological deficits through clinical exams and neurophysiological studies. The primary outcome is postoperatively hand function at three months. We anticipate a mean difference of 3 points with a standard deviation of 8 points, a power of 90%, and a type I error of 5%, resulting in a required sample size of 300 participants randomised 1:1. Secondary outcomes are neurological deficits (based on nerve conduction measurements, algometry test and von Frey hair test), clinical neurological examination of cutaneous sensibility, and registration of complications in the donor arm (haematoma formation, wound dehiscence, and/or infection).

The second comparison assesses two different proximal anastomotic sites, i.e. aorto-radial anastomosis versus mammario-radial anastomosis. The primary outcome is a composite of cerebrovascular events and the secondary outcome is graft patency evaluation by multi-slice computer tomography-scan. These outcomes will be assessed at 1 year postoperatively, and the results of this comparison will be exploratory only. Both comparisons will be analysed using intention-to-treat and intervention groups will be compared using linear regression, logistic regression, or Mann–Whitney *U* test depending on data type.

Two independent statisticians will follow the present plan and conduct the analyses which will hereafter be fused into a final analysis based on consensus.

**Conclusion:**

This detailed analysis plan will increase the validity of the NEO trial results by predefining the statistical analysis in detail.

**Trial registration:**

ClinicalTrials.gov identifier: NCT01848886. Registered 25 February 2013. Danish Ethics committee number: H-3–2012-116. Danish Data Protection Agency: 2007–58-0015/jr. n:30–0838.

**Supplementary Information:**

The online version contains supplementary material available at 10.1186/s13063-022-06869-7.

## Introduction

The NEO Trial is a randomised 2 × 2 factorial superiority clinical trial comparing endoscopic radial artery harvest (ERAH) versus open radial artery harvest (ORAH) (The NEO Trial 1) as well as comparing mammario-radial grafting (so-called Y-graft) versus aorto-radial grafting (The NEO Trial 2) [1]. All patients referred for coronary artery bypass grafting (CABG) at the Copenhagen University Hospital – Rigshospitalet, Denmark, were screened during the period May 2013 until October 2018. Informed consent was obtained from both elective and subacute CABG patients. A detailed protocol with trial background, design, and rationale has previously been published [1]. Overall, 300 participants were sequentially block-randomised into four intervention groups: (1) mammario-radial endoscopic group, (2) aorto-radial endoscopic group, (3) mammario-radial open surgery group, and (4) aorto-radial open surgery group.

## Methods

### Trial profile

The NEO Trial was registered at ClinicalTrials.gov (identifier: NCT01848886, 8 May 2013) prior to enrolment of the first participant and was carried out in compliance with The Helsinki Declaration [2]. The Committees on Biomedical Research Ethics of The Capital Region of Denmark (approval number: H-3–2012-116, 04 December 2012) and the Danish Data Protection Agency (approval number: 2007–58-0015/journal no. 30–0838, 23 August 2012) approved the NEO Trial.

**Fig. 1:**
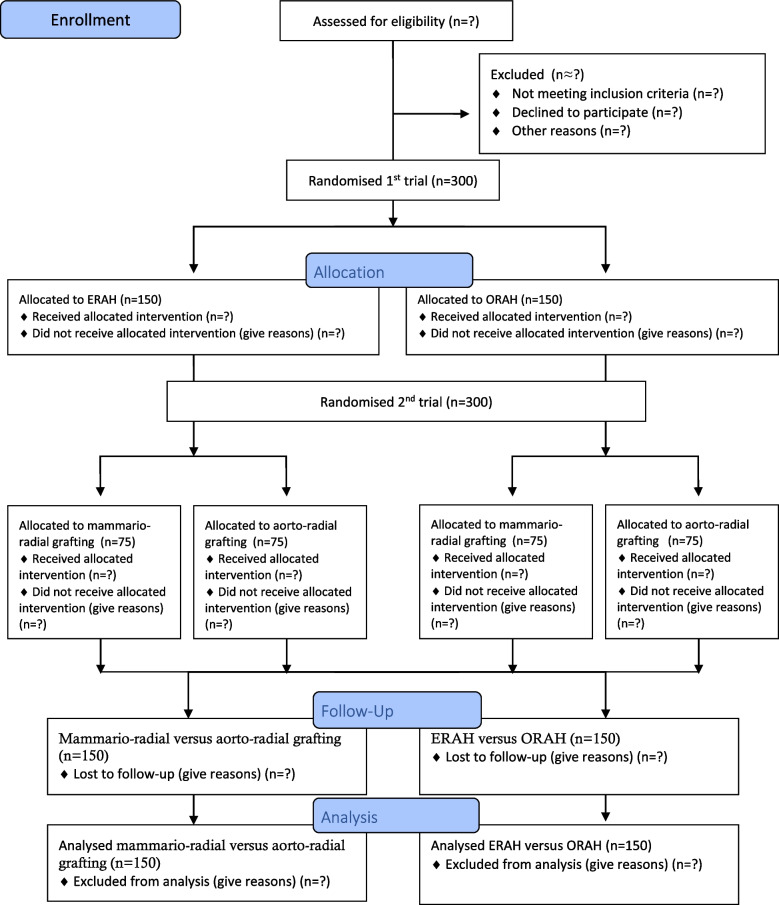
CONSORT flow diagram

As the trial is a 2 × 2 factorial designed trial, the outcomes are divided into two parts. All outcome measures are summarised in Table 1. The flow of trial patients will be displayed in a Consolidated Standards of Reporting Trials (CONSORT) diagram (Fig. 1).

**Table 1:** Outcome measures

	**Measurement time points** **(x = measuring point, X = outcome endpoint)**				**Data source**	**Blinding** **(Y = yes, N = no)**
	**Preoperatively**	**Before discharge**	**3 months**	**1 year**		
Hand function questionnaire	x	x	x	x	Questionnaire	N
Neurophysiological examination	x		x		Datasheet	Y
Clinical neurological examination	x		x	x	Case report form (CRF)	N
Complication rate		x	x		Database	Y
Serious adverse events		x	x	x	Register	Y
Scar evaluation			x	x	CRF	N
Handgrip strength	x	x	x	x	CRF	Y
Muscle function	x	x	x	x	CRF	Y
Vascular function	x		x		Datasheet	Y
Graft patency ERAH vs. ORAH				x	Datasheet	Y
Pain scale (LANSS)	x	x	x	x	CRF	N
Cardiac or cerebrovascular events		x	x	x	Register	Y
Graft patency free radial artery vs. Y-graft				x	Datasheet	N
Cutaneous sensation for cold	x	x	x	x	CRF	N
Neuropathy screening (UENS)	x				CRF	N
Demographic baseline data	x				CRF	N

### Inclusion and exclusion criteria

#### Inclusion criteria

- Elective/subacute CABG as an isolated procedure
- Age ≥ 18 years
- Multi-vessel coronary disease
- Non-dominant arm is eligible for radial artery harvest
- Written informed consent

#### Exclusion criteria

- Geographically not available for follow up
- Modified Allen’s test indicating insufficient ulnar artery perfusion [3].
- Valve surgery, ablation surgery, or any kind of concomitant surgery during same admission
- Acute operation (< 24 h from admission)
- Dialysis
- Preoperative neurological deficit in the donor arm
- Preoperative left ventricle ejection fraction (LVEF) < 20%
- Former sternotomy
- Contrast allergy
- Malignant disease
- No written informed consent

### Baseline characteristics

The baseline characteristics were assessed from inclusion in the trial until operation start. These characteristics will be:

1. Demographic characteristics:
  a Age
  b Sex (male, female)
  c Height
  d Weight

2. Preoperative status:
  a New York Heart Association (NYHA) functional classification of heart failure (I, II, III, IV, unknown) [4].
  b Canadian Cardiovascular Society (CCS) classification of angina pectoris (I, II, III, IV, unknown) [5].
  c Angina pectoris (yes, no)
  d Echocardiographic examination (left ventricular ejection fraction (LVEF), valvular dysfunction)
  e Recent acute myocardial infarction (AMI), i.e. within the last 90 days (yes, no)
  f Apoplexia cerebri or transitory cerebral attack (yes, no, year if yes, sequelae)
  g Nephrological status (creatinine, carbamide, glomerular filtration rate)
  h Pulmonary status (smoking history, pulmonary function test)
  i Gastrointestinal status (ulcus, liver cirrhosis, alcohol use)
  j Preoperative Euroscore I and II [6, 7]

3. Comorbidity
  a Hypertension (yes, no)
  b Hypercholesterolaemia (yes, no)
  c Diabetes (insulin dependent or non-insulin dependent)
  d Dialysis (yes, no)
  e Chronic obstructive pulmonary disease (COPD) (yes, no)

4. Surgical data:
  a Subacute or elective surgery

5. Screening for polyneuropathy:
  a Standardised amplitudes of motor and sensory nerve action potentials (*z*-scores) for the compared groups. These *z*-scores are diagnostic for polyneuropathy if two or more nerves (medianus, ulnaris, peroneus, or suralis) have a *z*-score of |*z*|≥ 2.

Baseline characteristics will be presented by intervention group. Discrete variables will be summarised by frequencies and percentages calculated according to the number of patients for whom data are available. Where values are missing, the actual denominator will be stated. Continuous variables will be summarised using standard measures of central tendency and dispersion, using either mean ± SD for data with normal distribution or median and interquartile range for non-normally distributed data. Tests for interaction between the interventions, each stratification, and design variables used to identify subgroups will be investigated only in an exploratory manner.

### Outcomes

The outcomes were defined as primary, secondary, and exploratory. Results will be published separately for the NEO Trials 1 and 2. All trial results and de-identified individual participant data whether statistically positive, negative, or neutral will end up in the public domain, preferably as peer-reviewed publications and in a public trial data repository. The preregistration at ClinicalTrials.gov (NCT01848886) and the published protocol [1] ensures the trial results are in accordance with CONSORT Statement [8]. An overview of outcomes can be seen in Table 1.

### Screening for polyneuropathy

All patients will undergo preoperative screening for poly-neuropathy. This will be done by a clinical examination using the Utah Early Neuropathy Scale (UENS) (Table 2) [9].

**Table 2:**
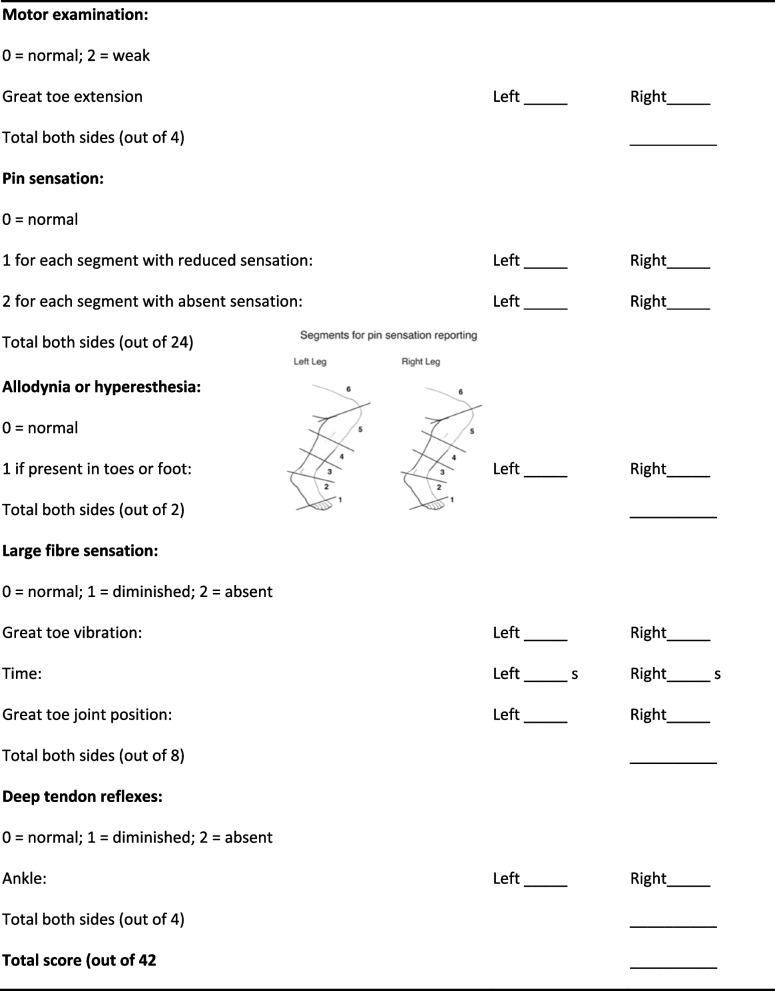
Utah Early Neuropathy Scale (UENS) [1]

UENS is a validated instrument of screening and grading neuropathy signs (0–42 score range) from the clinical neurological examination of the lower extremities in early peripheral neuropathy [9]. We control for bias from peripheral neuropathy between the study groups comparing the frequency of polyneuropathy (UENS Score > 3) and polyneuropathy load (ordinal outcome). We will further use the UENS score for exploratory examination of the correlation between polyneuropathy signs and the presence of compression neuropathy (carpal tunnel syndrome), presurgical amplitudes of motor and sensory nerve responses, and the change in amplitudes in the responses from the arm contralateral to the graft and the leg across the observation period. Thereby, UENS will be used to control for a bias between the two groups (ERAH versus ORAH) as to predisposition to a peripheral nerve lesion from a pre-existing polyneuropathy after randomisation (confounder control).

### The NEO Trial 1

The NEO Trial 1 compares the ERAH group versus the ORAH group.

#### Primary outcome

##### Hand function questionnaire

Postoperative questionnaire describing hand function at three months after randomisation. For each participant, the questionnaire results in a score between 5 and 49 points with 49 indicating worst outcome (see Supplementary material for a detailed description of all measurements).

#### Secondary outcomes

(A) Neurological deficit based on nerve conduction studies

Postoperative neurological deficits based on nerve conduction studies, monofilament test (von Frey test) of cutaneous sensibility, and pressure algometry of deep pain at 3 months after randomisation. For each participant, these outcomes will be classified as ‘deficit’ if one or more of the specific single measurements on the operated arm is below a predefined threshold, i.e. an increase in z-score ≥ 2 (Supplementary material).

(B) Neurological deficit based on clinical examination

Postoperative clinical deficits based on clinical examination at three months after randomisation. For each participant, cutaneous sensibility will be registered using a map of the forearm and hand with different colours spatially indicating a change in sensibility or occurrence of spontaneous pain sensation. The outcome will be classified as deficit if a change in colour occurs from no colour to any colour (Supplementary material).

(III) Complications in the donor arm

Postoperative complications that have occurred at three months after randomisation. For each participant, this outcome will be classified as a ‘complication’ if one of the mentioned complications occur (i.e. haematoma formation, wound dehiscence, and/or infection) within three months after randomisation (Supplementary material).

#### Exploratory outcomes

(A) Serious adverse events

 

The occurrence of serious adverse events registered at one year after randomisation. For each participant, this outcome will be classified as a ‘serious adverse event’ if one or more of the listed serious adverse events occur (i.e. reoperation for bleeding, revascularisation, myocardial infarction, stroke, or death) (Supplementary material).

(B) Scar evaluation

Scar evaluation at 1 year after randomisation.﻿ For each participant, this outcome will be assessed by a score from ‘0’ to ‘5’ with ‘5 being best scar result ﻿(Supplementary material).

(III) Handgrip strength

Hand grip strength at 1 year after randomisation. For each participant, this outcome will be measured using a hand dynamometer (Jamar Hydraulic Hand Dynamometer). The mean will be classified in 7 steps ﻿(Supplementary material).

(IV) Muscle power

Muscle power at one year after randomisation. For each participant, this outcome will be graded using the Oxford scale to grade strength in 6 different hand muscles. Grading consists of numbers from ‘0’ to ‘5 with ‘5’ being normal strength and a decrease in grading will be considered an occurrence of impairment ﻿(Supplementary material).

(E) Hand function questionnaire single items

Mean score of each of the hand function questionnaire items 2 through 9 (Table 3) at 3 months after randomisation. Individual participant data will consist of a number from 0 to 7 with 7 indicating worst outcome. Item 1 consist of dichotomous data (yes or no) and will be reported as such (Supplementary material).

**Table 3:** Hand function questionnaire [2]

**1. Right now, my hand and arm appear to be fine**	**6. I am concerned about the appearance of my arm scar**
(1) Yes	(0) No scar at all
(2) No	(1) No concern
**2. I feel pain in my arm or hand**	(2) Trivial concern
(1) No pain at all	(3) Mild
(2) Trivial	(4) Moderate
(3) Mild	(5) Quite concerned
(4) Moderate	(6) Very concerned
(5) Quite severe	(7) Extremely concerned
(6) Severe	**7. My arm has a scar that causes discomfort**
(7) Severe, unbearable pain	(0) No scar at all
**3. I feel numbness in my arm or hand**	(1) No discomfort
(1) No numbness at all	(2) Trivial discomfort
(2) Trivial	(3) Mild
(3) Mild	(4) Moderate
(4) Mode rate	(5) Quite uncomfortable
(5) Quite severe	(6) Very uncomfortable
(6) Severe	(7) Extremely uncomfortable
(7) Severe, unbearable numbness	**8. I have difficulties with daily tasks because of the use of my hand and arm**
**4. My arm or hand is swollen**	(1) No difficulties at all
(1) No swelling at all	(2) Trivial difficulties
(2) Trivial	(3) Mild
(3) Mild	(4) Moderate
(4) Moderate	(5) Quite marked
(5) Quite severe	(6) Very marked
(6) Severe	(7) Extremely marked
(7) Severe, unbearable swelling	Comments: ______________________
**5. I have limited use of my hand**	**9. Overall, my life is affected by the problems with my hand or arm**
(1) No limitations at all	(1) No worse at all
(2) Trivial	(2) Trivial life disruptions
(3) Mild	(3) Mild
(4) Moderate	(4) Moderate
(5) Quite severe	(5) Quite marked
(6) Severe	(6) Marked
(7) Extremely limited use	(7) Life radically worse
	Comments: ______________________

(F) Neurological deficits single tests

Postoperative neurological deficits based on nerve conduction studies, filament test of cutaneous sensibility, and pressure algometry of deep pain test at 3 months after randomisation. For each participant, this outcome will be classified as ‘deficit’ if any of the above tests meet their individual threshold for abnormality (Supplementary material).

(G) Multi-slice computed tomography (MSCT) evaluation of graft patency

Graft patency based on MSCT at 1 year after randomisation. For each participant, this outcome will be classified as ‘graft failure’ should incomplete patency, string sign, or occlusion occur (Supplementary material).

(H) Neuropathic pain symptoms and signs

The Leeds assessment of neuropathic symptoms and signs (LANNS) pain scale at three months after randomisation. For each participant, this outcome will be classified as ‘deficit’ if the test shows a cut-off value at ≥ 12﻿ (Supplementary material).

(I) Vascular function

Vascular function in the hand based on Technetium (^99m^Tc) sestamibi (MIBI) scan at three months after randomisation. For each participant, this outcome will be classified as ‘deficit’ using the ratio between thenar and hypothenar (Supplementary material).

### The NEO Trial 2

The NEO Trial 2 compares the mammario-radial group versus the aorto-radial group.

#### Primary outcome

##### Cardiovascular events

A composite outcome of all-cause mortality, myocardial infarction, target vessel revascularisation, or stroke at 1 year after randomisation (Supplementary material).

#### Exploratory outcome

(A) MSCT evaluation of graft patency

Graft patency based on MSCT at 1 year after randomisation. For each participant, this outcome will be classified as ‘graft failure’ should incomplete patency, string sign, or occlusion occur (Supplementary material).

### Data collection

Data was collected in the CRF preoperatively and postoperatively at 3 months and 1 year after randomisation. The CRF was paper-based and will be entered into a digital database (OpenClinica) by two independent investigators. Data will be collected from interviews and examinations of the participants, from the surgical database (named ‘PATS’), from the electronic patient journal system (named ‘Sundhedplatformen’ [10]), and from the Danish national patient registry (named ‘Landspatientregistret’ [11]).

The pre-planned 5- and 10-year follow-up will be analysed and reported as described for the 3 month and 1 year follow-up.

All data, including the trial master file and the statistical master file, will be handled centrally at the Copenhagen Trial Unit and will be stored on a secured server in a locked room. All data will be handled according to the Danish national legislation.

#### Timing

Before discharge, 3 months, and 1 year after the surgery, the participants were clinically evaluated for haematoma formation, infection, neurological deficits, and vascular dysfunction and the scar formation will be scored by a clinical examination. On the day before surgery and 3 months postoperatively, all participants underwent a motor and sensory nerve conduction study. A subgroup of 100 participants was selected randomly with 50% of patients in each of the ERAH and ORAH groups. This subgroup underwent physiological examination of vascular function in the hand preoperatively and 3 months after surgery. Before including the first participant, a pilot-study of five participants undergoing physiological examination of vascular function was conducted to evaluate examination technique implementation. In this physiological examination, the opposite non-operated arm will act as control. MSCT was conducted 1 year after surgery in all participants with blinded evaluation of the secondary outcome (graft patency evaluation by 320 slice-MSCT). Figure 1 shows a flowchart over the randomisation procedure used in this trial.

See Table 1 for the planned collection of outcome data.

#### Attrition

To avoid trial attrition, we have chosen short-term outcome of 1 year. The close and personal contact with the trial nurse also lessens risk of loss to follow-up. Trial participants will find it beneficial to follow 3 month and 1-year postoperative visits for optimal treatment and controls not offered to non-NEO trial participants. The trial nurse will directly contact participants if an outpatient visit is missed. Likewise, the trial nurse will keep contact information of the participants up-to-date after every contact.

### Blinding

The participants and surgeons cannot be blinded to the intervention used, as it will be obvious to both participants and surgeon whether the radial artery has been harvested with endoscopic or open techniques. Likewise, neither the patients nor the surgeons can be blinded to which proximal anastomotic site is used, since the surgeon will be performing the anastomosis and the participant has the right to be informed about the procedure performed.

The neurophysiology technicians cannot be blinded to whether ERAH or ORAH has been used since the neurophysiological examination requires placing electrodes near the scar evidently showing which procedure was used.

The NEO trial nurse cannot be blinded since she will be aware of screening, randomisation, and outcome measurements of the individual patients.

The clinical staff examining handgrip strength and muscle function will be blinded to which intervention has been used.

The staff interpreting the MSCTs cannot be blinded in consideration to the NEO Trial 2 but will be blinded in consideration to the NEO Trial 1.

The data collection will be blinded when using register data.

The statistical analysis of the trial will be blinded with the intervention groups coded as, e.g. ‘X’ and ‘Y’, following which two conclusions will be drawn: one assuming ‘X’ is the experimental group and ‘Y’ is the control group, and one conclusion assuming the opposite. After this, the blinding will be broken.

### Calculation of sample size

We planned a trial of an approximated continuous response variable (hand function) from independent control and experimental participants with approximately one control per experimental participant. In a previous study, the response within each participant group was normally distributed with standard deviation of 8 [12]. If the true difference in the experimental and control means was 3, we would need to study 150 experimental participants and 150 control participants to be able to reject the null hypothesis that the population means of the experimental and control groups would be equal with a probability (power) 90%. The type I error probability associated with this test of this null hypothesis was 5%. In total, we thus needed to include 300 participants.

### Power estimations of non-primary outcomes

The following will describe power estimations for secondary outcomes in the NEO Trial 1.

(A) Neurological deficit based on nerve conduction studies

Assuming a difference in occurrences of neurological deficits of 30% in the experimental group versus 15% in the control group, using a type I error of 5% and by including 300 participants, we will have 88% power to detect the difference between the two groups.

(B) Neurological deficit based on clinical examination

Assuming a difference in cutaneous sensibility of 30% in the experimental group versus 15% in the control group, using a type I error of 5% and by including 300 participants, we will have 88% power to detect the difference between the two groups.

(III) Complications in the donor arm

Assuming a difference in complications of 7% in the experimental group versus 1% in the control group, using a type I error of 5% and by including 300 participants, we will have 76% power to detect the difference between the two groups.

Power estimations for exploratory outcomes.

The mentioned outcomes are ‘exploratory’, as we have not been able to perform power calculations due to none or very limited data from previous trials or studies.

### General statistical analysis

Statistical analyses will be conducted blinded and independently by two statisticians from the Copenhagen Trial Unit according to this detailed statistical analysis plan. No interim analysis was planned. A statistical analysis plan (SAP) was planned to be published before initiation of analyses following the reporting guidelines [13].

Intention-to-treat analysis (ITT) will be used. Our primary conclusions will be based on the primary outcome of the NEO Trial 1. The results of the NEO Trial 2 are expected to be underpowered and will be reported for hypothesis-generating purposes only. Adherence to the intervention will be reported narratively.

#### Stratification and design variables

The primary analysis will for all outcomes except count data outcomes be adjusted for the stratification variables (age and sex). Age will be divided into two groups: (1) age up to 59 years and (2) age 60 years and older. Stratification according to sex will divide into two groups (1) male and (2) female sex.

#### Analysis of continuous outcomes

Continuous outcomes will be described as mean, mean difference, standard deviation, and 95% confidence intervals. Differences between the groups will be compared using linear regression.

#### Analysis of dichotomous outcomes

Dichotomous outcomes will be summarised as numbers, percentages, relative risks, and 95% confidence intervals. Logistic regression will be used to compare the intervention groups. We will estimate relative risks using Stata ‘nlcom’ command.

#### Analysis of count data outcomes

Count data outcomes will be summarised as numbers and percentages, medians, and interquartile ranges. Differences between groups will compared using Mann–Whitney *U* test and Hodges–Lehmann median differences (difference between participants and not difference between groups), and confidence intervals will be reported.

#### Threshold of significance

The thresholds for significance will be assessed according to the 5-point procedure suggested by Jakobsen et al. [14]. We will report exact p-values and report 95% confidence intervals for the outcomes. Neither the primary nor secondary endpoints will be corrected for multiplicity.

#### Missing data

Missing data will be handled according to the recommendations by Jakobsen et al. [3]. In brief, we will consider using multiple imputation and present worst-best and best–worst case scenarios, where all missing data is added as both the best case and worst case scenario. This will only be carried out if the missing data cannot be ignored. The complete case analysis will be considered the primary analysis and the remaining analyses (analysis based on multiple imputation/the worst-best and best–worst analyses) will be consdidered sensitivity analyses. We will in detail report if data are missing and why (withdrawal of informed consent, lost to follow-up, etc.).

### Outline of figures and tables

Figures and tables will include:

- CONSORT flow chart (Fig. 1)
- Table of measurement points (Table 1)
- Kaplan–Meier plot after 1 year comparing the 2 × 2 groups
- Baseline characteristic tables for the 2 × 2 groups
- Tables of graft patency (ORAH vs ERAH, aorto-radial versus mammario-radial)
- Tables of postoperative complications divided into groups
- Tables of Technetium-99 m sestamibi (MIBI) scan results
- Specific tables with the results of neurophysiological and clinical exams

### Assessments of underlying statistical assumptions

We will systematically assess underlying statistical assumptions for all statistical analyses according to the recommendation by Jakobsen et al. [15, 16]. In short, for *all* regression analyses, both primary and secondary, we will test for major interactions between each covariate and the intervention variable. We will, in turn, include each possible first order interaction between included covariates and the intervention variable. For each combination, we will test if the interaction term is significant and assess the effect size. We will only consider that there is evidence of an interaction if the interaction is statistically significant after Bonferroni adjusted thresholds (0.05 divided by number of possible interactions and if the interaction shows a clinically important effect). If it is concluded that the interaction is significant, we will consider both presenting an analysis separately for each [15, 16].

#### Assessments of underlying statistical assumptions for continuous outcomes

We will visually inspect quantile–quantile plots of the residuals [17, 18] to assess if the residuals are normally distributed and use residuals plotted against covariates and fitted values [17, 18] to assess for homogeneity of variances. If the plots show deviations from the model assumptions, we will consider transforming the outcome, e.g. using log transformation or square root and/or use robust standard errors [16–18].

#### Assessments of underlying statistical assumptions for dichotomous outcomes

We will assess if the deviance divided by the degrees of freedom is significantly larger than 1 to assess for relevant overdispersion. Overdispersion is the presence of greater variability (statistical dispersion) in a data set than would be expected based on a given statistical model, and in this case consider using a maximum likelihood estimate of the dispersion parameter.

### Statistical reports

Blinded data on all outcomes will be analysed by two independent statisticians [16]. Two independent statistical reports will be sent to the principal investigator and will be shared with the steering group and author group, and if there are discrepancies between the two primary statistical reports, then possible reasons for that will be identified and the steering group will decide which is the most correct result. A final statistical report will be prepared, and all three statistical reports will be published as supplementary material [16].

## Discussion

This article describes the detailed statistical analysis plan for the NEO Trials 1 and 2 in order to avoid risks of outcome reporting bias and data-driven results. The plan is to report primary and secondary results in separate publications and the exploratory outcomes in multiple thematic publications.

We will analyse data in accordance to the intention-to-treat principle. If necessary, we will use multiple imputations and best–worst/worst/best case scenario to assess the potential impact of the missing data on the results.

This statistical analysis plan is based on the trials primary protocol but has been some time in the making. The large number of outcomes measured as well as the complexity of the measurements conducted greatly inflicted the delay of the article. Together with the worldwide increased and changed workload during the last 2 years due to COVID-19, it has been difficult to engage the trial group in non-clinical work to finish this article. In order to secure against trial bias, the last CRF data has yet to be entered into the trial database and no data has been retrieved nor analysed.

### Strengths

The NEO trial will be able to compare the endoscopic versus the open surgery radial artery harvesting techniques. Only six smaller randomised trials have been conducted trying to assess the patency and complications in ERAH versus ORAH [19–24]. The NEO Trial 1 will be able to assess if there are significantly less neurological complications when harvesting the radial artery with an endoscopic technique than by an open technique. No significant decrease in the vascular supply in the donor arms compared to non-donor arms has been indicated in previous studies [25, 26], but the NEO Trial 1 will try to assess if there could be a relative ischaemic state after radial artery harvest using a new diagnostic test. Particularly, any neurological deficits can limit the hand function of the patients postoperatively, and it is important to try to get a clearer picture of this. The NEO Trial 1 will also examine if there is any difference in patency in ERAH participants versus ORAH participants. This is an important point to be addressed since, if there is any difference, it can have substantial consequences for the patients. All these points of investigation put together will determine the future of endoscopic harvest of the radial artery. We believe and hypothesise that the technique will be beneficial to the patients and will render the endoscopic procedure as the preferred technique when harvesting the radial artery for arterial revascularisation.

Another point of interest that the NEO Trial 2 tries to enlighten is the optimal site of the proximal anastomosis when using the radial artery as a bypass graft. Previously, no randomised clinical trial has assessed if aorto-radial or mammario-radial anastomosis is the best choice. We are well aware that the NEO Trial 2 does not have sufficient power to show a significant difference when measuring major cerebrovascular events, we hope to get enough data to find a likely sample size for another randomised trial dedicated to this question. If this trial shows indications of the mammario-radial technique being non-inferior to the aorto-radial technique, it will inform future research. The mammario-radial technique has the strength of avoiding the side-clamp and the possibility to revascularise all three coronary vessels with a minimum of grafts required.

Another strength of the NEO Trial is the 2 × 2 factorial design. This enables the trial to examine the benefits and harms of two different surgical strategies in the setting of one randomised clinical trial. The complexity and elaborate neurological examinations including both objective tests and subjective answers on questionnaires is also a strength of the NEO trial. This will make us able to both evaluate patient related parameters, such as quality of life, as well as factual complications comparing ORAH versus ERAH. In an evolving field of modern cardiac surgery using arterial revascularisation and endoscopic technique, this trial will contribute with important facts necessary for optimising patient treatment.

### Limitations

Our trial also has limitations. Neither the surgeon nor the patients can be blinded as to which operational technique has been used. However, all outcome assessments available for blinding will be blinded. There will be an evaluator risk of bias in the NEO Trial 1 objectives as the trial nurse cannot be blinded. Our trial is designed as a 2 × 2 factorial design, and we assume that there are no significant interactions between the different trial interventions. Lack of power may hinder confirmation or rejection of this assumption which is a further limitation.

## Conclusions

This article describes the planned detailed statistical analyses for publication of NEO Trials 1 and 2 outcomes in order to minimise risk of reporting bias and data-driven results.

## Supplementary Information

**Additional file 1.** Statistical Analysis Plan (SAP) Checklist.

**Additional file 2.** Supplemental material on outcomes.

## Data Availability

The datasets during and/or analysed during the current study available from the corresponding author on reasonable request.

## Abbreviations

CABG: Coronary artery bypass grafting surgery
CCS: Canadian Cardiovascular Society classification of angina pectoris
cMAP: Compound motor action potential
CRF: Case report form
ECC: Extracorporeal circulation
ERAH: Endoscopic radial artery harvest
EVH: Endoscopic vein harvest
LAD: Left anterior descending artery
LIMA: Left internal mammary artery
MSCT: Multi-slice computed tomography
NEO: Neurological complications in endoscopic versus open radial artery harvest
NYHA: New York Heart Association functional classification of heart failure
ORAH: Open radial artery harvest
PDA: Posterior descending artery
SNAP: Sensory nerve action potential
SPECT: Single photon emission computed tomography
