# Supplementary material for: Detailed statistical analysis plan for the neurological complications in endoscopic versus open radial artery harvest (NEO) randomised clinical trial
Source: Trials. 2022 Dec 9;23:990. doi: 10.1186/s13063-022-06869-7 (PMC9734608; doi:10.1186/s13063-022-06869-7)
Supplement: Supplementary file 2 — Additional file 2. Supplemental material on outcomes. [file 13063_2022_6869_MOESM2_ESM.docx]

Detailed statistical analysis plan for the Neurological complications in Endoscopic versus Open radial artery harvest (NEO) randomised clinical trial - Supplemental material on outcomes

### Authors

Christian L. Carranza^a^, Martin Ballegaard^c^, Mads U. Werner^d^, Philip Hasbak^e^, Andreas Kjaer^e^, Klaus Kofoed^f^, Markus Harboe Olsen^b,g^, Christian Gluud^b,h^ and Janus Christian Jakobsen^b,h^

^a^ Department of Cardio-thoracic Surgery, The Heart Centre, Copenhagen University Hospital − Rigshospitalet, Copenhagen, Denmark.

^b^ Copenhagen Trial Unit, Centre for Clinical Intervention Research, The Capital Region, Copenhagen University Hospital − Rigshospitalet, Copenhagen, Denmark.

^c^ Department of Neurology, Zealand University Hospital, Roskilde, Denmark

^d^ Multidisciplinary Pain Centre, Department of Anesthesia, Pain and Respiratory Support, Neuroscience Center, Copenhagen University Hospital − Rigshospitalet, Copenhagen, Denmark.

^e^ Department of Clinical Physiology, Nuclear Medicine and PET and Cluster for Molecular Imaging, Copenhagen University Hospital – Rigshospitalet and Department of Biomedical Sciences, University of Copenhagen, Denmark.

^f^ Department of Cardiology and Radiology, Copenhagen University Hospital − Rigshospitalet, Copenhagen, Denmark.

^g^ Department of Neuroanaesthesiology, The Neuroscience Centre, Copenhagen University Hospital − Rigshospitalet, Copenhagen, Denmark.

^h^ Department of Regional Health Research, The Faculty of Health Sciences, University of Southern Denmark, Odense, Denmark.

**Correspondence:**

Christian Lildal Carranza, MD, E-MBA, mail: christian@thoraxkir.dk, phone: +45 35451488

Department of Cardio-thoracic Surgery, The Heart Centre, Copenhagen University Hospital − Rigshospitalet, Blegdamsvej 9, DK-2100 Copenhagen Ø, Denmark.

## NEO Trial 1 outcomes

### Primary outcome

#### Hand function questionnaire

Sum score of hand function questionnaire (Table 3) items 2 through 8^1^. The mean values in the ERAH group will be compared with the mean value in the ORAH group at three months after surgery. Data consists of number from 5 to 49 points with 49 indicating worst outcome.

The hand function questionnaire has been proposed by the RAPCO study as a tool to evaluate quality of life impact by radial artery harvest^1^. This outcome will be non-blinded. The trial nurse assigned to the NEO trial will record the responses of the participants. Source data will be the completed questionnaire.

###### Sample size estimations for primary outcome

In NEO Trial 1, we are comparing endoscopic (experimental) with open (control) technique when harvesting the radial artery. Used in a previous trial, the data for each of the two groups, when assessing the score on the hand function questionnaire, was normally distributed with a standard deviation of 8^1^. If the true difference in the experimental and control means is 3, we estimated that we would need to study 150 experimental participants and 150 control participants to be able to reject the null hypothesis that the population means of the experimental and control groups are equal with probability (power) 90%^2^. The type I error probability associated with this test of this null hypothesis is 5%. In total, we thus need to include 300 participants.

### Secondary outcomes

#### (A) Neurological deficit based on nerve conduction studies

The nerve function will be assessed in joint venture with the Department of Neurophysiology, Rigshospitalet, Copenhagen, Denmark, and is planned as follows:

| 1. | Cutaneous sensibility on both forearms and hands by appraisal of dermatomes. All sensibility modalities are examined:   \| a. \| Cutaneous touch sensibility examined by von Frey filaments. \| \| --- \| --- \| \| b. \| Deep pain sensibility examined by pressure algometry. \| |
| --- | --- | --- | --- | --- | --- |
| 2. | Sensory nerve conduction studies performed on both arms:   \| a. \| Median nerve (digit 2 – wrist); orthodromic technique. \| \| --- \| --- \| \| b. \| Ulnar nerve (digit 5 – wrist); orthodromic technique. \| \| c. \| Superficial sensory branch of the radial nerves (forearm – wrist); antidromic technique. \| \| d. \| Lateral antebrachial cutaneous nerve (forearm – elbow); antidromic technique. \| \| e. \| Medial antebrachial cutaneous nerve (forearm – elbow); antidromic technique. \| |
| 3. | Motor nerve conduction studies on both arms:   \| a. \| Median nerve (wrist – abductor pollicis brevis muscle (APB), elbow – APB). \| \| --- \| --- \| \| b. \| Ulnar nerve (wrist – abductor digiti minimi muscle). \| |
| 4. | Control nerve conduction studies on legs:   \| a. \| Sural nerve dexter, sensory nerve conduction study; antidromic technique. \| \| --- \| --- \| \| b. \| Peroneal nerve dexter, motor nerve conduction study \| |

Neurophysiology technicians will perform the nerve conduction studies and the trial nurse will perform the quantitative sensory testing.

The outcome measure will be a composite of the 9 different tests performed. When > 1 of these tests deteriorates significantly from preoperative values, the conclusion will be that neurological damage has taken place. The patients will undergo full examination preoperatively and at 3 months. Both arms are tested but only a significant change in the operated arm will be registered as related to the intervention as long as there is no damage on the non-operated arm. Otherwise, the participant will be registered with possible systemic neurological damage.

| The three different exam types are as follows:  • von Frey filaments: measurement unit is milliNewton (mN). The filaments are individually calibrated to a tolerance range of ±10% of its nominal force. The standard set includes 12 monofilaments (corresponding to 12 successively increasing nominal values) in the range of 0.25 to 512 mN with the force increasing by a factor of two from filament to filament. |
| --- |
| A significant clinical deterioration is from one nominal value to ≥1 nominal value assessed as mean values at five sites on the hand and forearm. Both arms are tested but only a significant change in the operated arm will be registered as related to the intervention as long as there is no damage on the control, the non-operated arm. Otherwise, the participant will be registered with possible systemic neurological damage. The five sites are as follows:   - Thenar (lateral part) - Palmar base of digiti manus III - Palmar base of digiti manus V - Lateral part of the antebrachium - Medial part of the antebrachium   • Algometry test: A pressure algometer (application surface 1 cm^2^) is placed over the palmar base of metacarpal II on the donor arm and pressure is applied (20 to 40 kiloPascal (kPa)/s) until the pain threshold is reached. Corresponding control-assessments are made contralaterally over the palmar base of metacarpal II. The test is validated for pain assessments over tender-points in normal muscles^3^. |
| A significant deterioration is a change in pressure pain threshold of ≥ 50 kPa (if the measurement is < 150 kPa) or ≥ 100 kPa (if the measurement is ≥ 150 kPa). If a significant deterioration also occurs in non-donor arm the patient will be registered with possible systemic neurological damage.  • Sensory nerve conduction study: The peripheral nerves are activated using a surface stimulation electrode and the resulting sensory nerve action potential (SNAP) is recorded with surface electrodes over the nerve at a point more distal than or proximal to the stimulation point. The latency and amplitude of the SNAP is measured and the conduction velocity is calculated using the distance between stimulation and recording point. The results are compared to national multi-centre age-controlled normative values and reported as z-scores. |
| • Motor nerve conduction studies: The compound motor action potential (cMAP) is recorded from the muscle belly using surface electrodes in a pseudomonopolar configuration. The active electrode is positioned over the motor point of the muscle belly and the reference electrode is positioned at a nearby inactive site according to the laboratory procedures. The electrodes are readjusted to result in a cMAP with an initial negative deflection and a maximal amplitude. Responses from supramaximal stimulation at the wrist and in the median nerve also at the elbow are recorded. Distal motor latency and amplitude of the maximal cMAP is measured and motor nerve conduction velocity is calculated from the distance between stimulation sites. The results are compared to national multi-centre age-controlled normative values and reported as z-scores. |

In both sensory and motor nerve conduction studies, significant changes in amplitudes of the responses are defined as a deterioration of any amplitude > 2 z-score points. Seven neurophysiological exams are clinically related to the intervention, but two supplemental exams (one sensory and one motor) are done on the leg. If a change also occurs at these locations the damage will be uncorrelated to the intervention and the participant will be registered with possible systemic neurological damage.

The outcomes will be non-blinded. The neurophysiology technician will not be blinded towards knowledge regarding the use of an endoscopic or open technique, since it will be evident by the scarring. Source data will be the standardised examination datasheet from the Department of Neurophysiology and the case report form (CRF) used by the trial nurse. As neurophysiological non velocity testing is sensitive to room temperature and relative humidity this is registered in the CRF at every neurophysiological non velocity test visits.

#### (B) Neurological deficit based on clinical examination

Change in cutaneous sensibility from baseline to the time point 3 months after surgery. Subjective changes in cutaneous sensibility will be registered using a map of the forearm and hand on which the participant can note changes coded by colour. The change values of the donor arm in the ERAH and ORAH group will be compared. A significant change is one from no colour to any colour (figure 2 for spatial dimension). Mean total colour change between the groups will be reported as well as individual colour changes between the groups. Colour coding is as follows:

- No colour = normal sensibility
- Blue colour = no sensibility or decrease sensibility
- Green colour = discomfort or changed sensibility
- Red colour = spontaneous pain sensation

The trial nurse will, together with the participants, evaluate the change in cutaneous sensibility using a map of the forearm and hand to mark sensibility changes in the operated forearm and hand. Cutaneous sensibility will also be evaluated on non-donor arm to ensure test reliability and to test for mirror-image-defects post-surgery. The subjective perception intensity necessary to define the four groups of described sensibilities are patients’ choice.

This outcome will be non-blinded. The trial nurse assigned to the NEO trial will be trained by the authors to perform the clinical neurological examinations. Source data will be the CRF used by the trial nurse.

#### (C) Complications in the donor arm

Occurrence of complications at 3 months after surgery. Complications are defined as a composite of haematoma formation, wound dehiscence, or infection registered postoperatively (before discharge) and 3 months after the operation by the trial nurse.

This outcome will be non-blinded. The trial nurse assigned to the NEO trial will register the complications occurring in the operated sites of ERAH and ORAH participants. Source data will be database data from the surgical complication register.

### Exploratory outcomes

#### (A) Serious adverse events

Occurrence of the following serious adverse events at one year after surgery: reoperation for bleeding; revascularisation; myocardial infarction; stroke or death.

Data will consist of mentioned events and the results will be compared between ERAH versus ORAH groups.

This outcome will be blinded. The data will be collected directly from the Danish Patient Register using the participant’s civil registration number.

#### (B) Scar evaluation

Stony Brooke Scar Evaluation Score^4^ (Table 4) at one year after surgery.

Data will consist of scores ranging from “0” to “5” with “0” signifying worst and “5” signifying best scar result. Comparison of mean scores in the ERAH and the ORAH group will be done.

This outcome will be non-blinded. The trial nurse assigned to the NEO trial will be trained in and perform the scar evaluation. The data source will be the CRF.

#### (C) Handgrip strength

Maximal handgrip strength one year postoperatively (Table 5).

A hand dynamometer will be used to measure the maximum isometric hand and forearm muscle strength on both hands with the elbows flexed. Three consecutive tests will be done with 15 seconds of recovery time between tests; this test has been validated^5^. Measuring unit is kilograms and rating of the test is seen in Table 5.

Data will consist of a mean of three tests in both arms. The mean will be rated in seven steps from worst to best. Comparison of mean rates between the ERAH and the ORAH group will be done.

This outcome will be semi-blinded. An independent health care professional who performs the handgrip strength measurements will be blinded by covering the skin of the donor arm to disguise the harvest technique used. The data source will be the CRF.

#### (D) Muscle power

The following muscles will be rated according to the Oxford Scale for grading muscle strength (Table 6) at one year postoperatively: abductor pollicis brevis muscle; abductor digiti minimi muscle; 1^st^ interosseus dorsalis muscle; flexor digitorum profundus muscle to finger 2 and 5; and extensor digitorum communis muscle.

The rating scale is sometimes also referred to as ‘Medical Research Council Scale for Muscle Strength’. The grading consists of numbers from “0” to “5” with “5” being normal strength. A decrease in grading comparing pre-operative with postoperative will be considered a significant change. Thereafter comparison of total occurrence as well as individual muscle impairment occurrence will be done between the ERAH and ORAH group.

This outcome will be semi-blinded. An independent health care professional will perform the muscle strength evaluation blinded by covering the donor arm to disguise the harvest technique used. The data source will be the CRF.

#### (E) Hand function questionnaire single items

Mean score of each of the hand function questionnaire items 2 through 9 (Table 3). The mean values in the ERAH group will be compared to the mean value in the ORAH group at 3 months after surgery. Individual participant data will consist of a number from 0 to 7 with 7 begin worst outcome. Item 1 consist of dichotomous data (yes or no) and will be reported as such between the ERAH and ORAH group.

#### (F) Neurological deficits single tests

Occurrences of neurological deficits at 3 months after surgery. The deterioration of each of the clinical neurological tests is defined as a secondary outcome. The neurological deficits consist of following types of single tests:

• Neurophysiological examination: Nine different nerves are neurophysiologically assessed and an increase in z-score >2 is a significant change. Seven peripheral nerves innervating the arms (clinically relevant outcome) and two innervating the legs (control of possible systemic neurological damage i.e., nonintervention relevant deterioration) are examined. Total occurrence of nerve damage as well as individual nerve damage (seven test points) will be reported comparing ERAH group and ORAH group.

• von Frey filament examination: Five different sites are tested (mentioned earlier) and a nominal change of ≥ 1 filament size is a significant change. The range is 12 filament sizes and a mean of five tests performed are the final median force registered. This is done with both arms and if a significant change takes places in non-operated arm the patient will be excluded from intervention related damage, but will still be reported as a possible occurrence of systemic neuropathy in the trial population.

• Algometry test: One test at the palmar base of metacarpal II donor arm as well as same test site on non-operated arm are tested.

The outcomes will be non-blinded. The neurophysiology technician will not be blinded towards knowledge regarding the use of an endoscopic or open technique, since it will be evident by the scarring. Source data will be the standardised examination datasheet from the Department of Neurophysiology and the case report form (CRF) used by the trial nurse. As neurophysiological non velocity testing is sensible to room temperature and humidity this is registered in the CRF at every neurophysiological non velocity test visits.

#### (G) MSCT evaluation of graft patency

Patency of the graft at one year after the surgery. The patency will be divided into perfect patency, incomplete patency, string sign, and occluded according to assessment by MSCT, and the ERAH will be compared to the ORAH group both in total graft incompleteness (sum of incomplete patency, string sign and occlusion) and by individual results. The NEO Trial 1 part will only look at the anastomosis between the radial artery and the coronaries. Therefore, patients with a malfunctioning mammario-radial anastomosis will be excluded from this analysis.

The MSCT allows for assessment of cardiac structures and both 16-slice and 64-slice scanners have been validated for assessing the graft patency after CABG [57,58]. The patients included in the study are scanned using a 320-slice scanner (Toshiba Aquilion ONE, Japan). The scanning protocol is as follows: Gantry rotation time 350 ms, detector collimation 0.5 × 320. Tube voltage and current are chosen based on the patient’s body mass index ranging between 100 and 120 kV and between 280 and 500 mA. An intravenous contrast media (Visipaque 320 mg/ml, GE Healthcare, UK) is infused using a flow rate of 6 ml/s followed by a saline chaser. The contrast dye volume used is individually calculated according to patient body mass index (100–130 ml). Image interpretation is performed using commercially available software (Vitrea, version 3.0.1, Vital Images, USA). Grafts are evaluated by two experts in cardiac MSCT blinded to the patient’s randomisation status in the trial. MSCT is performed at the department of radiology one year after the operation.

This outcome will be blinded. Two employees from the Department of Cardiology and Radiology will do the interpretation of MSCT scans for patency, without knowledge of whether the radial artery has been harvested by endoscopic or open techniques.

#### (H) Neuropathic pain symptoms and signs

The Leeds assessment of neuropathic symptoms and signs (LANNS) pain scale^6^ at 3 months after surgery (Table 7). The Danish version of this assessment is tested to have a cut-off value at ≥12.

This outcome will be non-blinded. The trial nurse assigned to the NEO trial will record the responses of the participants. The data source will be the CRF.

#### (I) Vascular function

The vascular function will be assessed by single photon emission computed tomography (SPECT) of the hand. The technique used is ^99m^Technetium sestamibi imaging which is commonly known as a ‘MIBI scan’ and is most often used for myocardial perfusion^7^. ^99m^Technetium sestamibi is a lipophilic cation which when injected distributes accordingly to the blood perfusion. Using a gamma camera, it is possible to register the gamma rays emitted by the decay of ^99m^Technetium sestamibi. When injected after stress exercise a possible insufficiently perfused area will be evident.

A total of 100 patients will be randomly included in the ^99m^Technetium sestamibi imaging examination of the hand; 50 patients will come from each of the ERAH and ORAH groups. There will be no consideration taken as to whether they are part of the aorto-radial or the mammario-radial groups. These patients will preoperatively and 3 months postoperatively be examined for MIBI estimated perfusion in the donor hand comparing 3-month values to baseline values. Values will also be compared to the non-donor hand. The MIBI scan will show if there is any difference in perfusion after removal of the radial artery. There have been no previous studies examining the hand with MIBI scans, so the examinations aim to validate the technique in this setting. We propose that the activity accumulation (counts/cm^2^) ratio between measurements on thenar (base of the thumb) and on hypothenar (base of the little finger) can be a reliable indicator of hypoperfusion in the hand after removal of the radial artery.

Staff employed at The Department of Clinical Physiology, Nuclear Medicine, & PET, will perform the MIBI scans.

Outcome measure will be the quantitative difference in MIBI-scan estimated perfusion in the donor and non-donor hand. Time point will be 3 months after surgery. The measuring unit is counts/cm^2^ that may be converted to estimate of Bq/cm^2^.

Since no previous similar test has been found in the literature search it is not possible to do pretrial power calculations.

This outcome will be blinded. The Department of Clinical Physiology, Nuclear Medicine, & PET will perform the exams and interpretations without knowledge of whether the radial artery has been harvested by endoscopic or open techniques. The data source will be a datasheet from the Department of Clinical Physiology.

## NEO Trial 2

### Primary outcome

#### Cardiovascular events

Occurrence of one of the following cardio- or cerebrovascular events: all-cause mortality, myocardial infarction, target vessel revascularisation, or stroke at one year postoperatively.

The outcome will be blinded. The data will be collected directly from the Danish Patient Register using the participant’s social security number for those patients lost to clinical follow-up. Patients completing their clinical visits will have these occurrences registered on the CRF. We are well aware of the exploratory nature of this outcome (please see below).

###### Power estimations for primary outcomes

In the other part of the trial, we are comparing mammario-radial (experimental) with aorto-radial (control) grafting. One case-control study found an event-free survival at 12 months of 97% using arterial revascularisation compared to 67% using venous revascularisation^8^. Another case-control study showed a survival free of cardiac event or death at 2.5 years of 11% for free grafts versus 17% for Y-grafts, but the risk of re-angina was 6.6% in the free graft group versus 4.6% in the Y-graft group^9^. No studies showed exactly what difference in occurrence of cerebrovascular composite outcomes could be expected between free grafts and Y-grafts, but a randomised clinical trial comparing arterial Y-graft with free saphenous vein grafts found significantly lower cardiac event-free survival at less than 2 years with a difference of about 20% events in free grafts versus 5% in Y-grafts^8^. Considering these studies, we expect maximally a difference in occurrence of cardio or cerebrovascular outcomes at one year postoperatively of 5% (15% in mammario-radial group vs. 10% in aorto-radial group).

We are planning a trial with approximately 150 experimental participants and 150 control participants. We will then only have a power of 25.7% to detect the difference of 15% composite outcomes in mammario-radial group versus 10% in aorto-radial group using a type I error probability of 5%. We will use an uncorrected χ^2^ statistic to evaluate this null hypothesis. Therefore, the NEO trial 2 will only be an exploratory trial to plan the size of a future randomised trial concerning the occurrence of cardiac and cerebrovascular events using mammario-radial versus aorto-radial anastomosis.

### Exploratory outcome

#### (A) MSCT evaluation of graft patency

Same technique and procedure will be used as mentioned earlier in the text.

Outcome measure will be patency of the graft at one year after surgery. The patency will be divided into perfect patency, occluded, incomplete patency, and string sign according to assessment by MSCT.

The outcome will be non-blinded. Two employees from the Department of Cardiology and Radiology will do the interpretation of MSCT scans for patency, but it will be evident on the MSCT which proximal anastomosis site is used.

## **References**

1. Zhu YY, Hayward PAR, Hadinata IE, et al. Long-term impact of radial artery harvest on forearm function and symptoms: A comparison with leg vein. *J Thorac Cardiovasc Surg*. 2013;145(2):412-419. doi:10.1016/j.jtcvs.2012.01.052

2. Carranza CL, Ballegaard M, Werner MU, et al. Endoscopic versus open radial artery harvest and mammario-radial versus aorto-radial grafting in patients undergoing coronary artery bypass surgery: protocol for the 2 × 2 factorial designed randomised NEO trial. *Trials*. 2014;15:135. doi:10.1186/1745-6215-15-135

3. Fischer AA. Pressure algometry over normal muscles. Standard values, validity and reproducibility of pressure threshold. *Pain*. 1987;30(1):115-126.

4. Singer AJ, Arora B, Dagum A, Valentine S, Hollander JE. Development and validation of a novel scar evaluation scale. *Plast Reconstr Surg*. 2007;120(7):1892-1897. doi:10.1097/01.prs.0000287275.15511.10

5. Reuter SE, Massy-Westropp N, Evans AM. Reliability and validity of indices of hand-grip strength and endurance. *Aust Occup Ther J*. 2011;58(2):82-87. doi:10.1111/j.1440-1630.2010.00888.x

6. Bennett M. The LANSS Pain Scale: the Leeds assessment of neuropathic symptoms and signs. *Pain*. 2001;92(1-2):147-157.

7. Hesse B, Tägil K, Cuocolo A, et al. EANM/ESC procedural guidelines for myocardial perfusion imaging in nuclear cardiology. *Eur J Nucl Med Mol Imaging*. 2005;32(7):855-897. doi:10.1007/s00259-005-1779-y

8. Muneretto C, Bisleri G, Negri A, et al. Left internal thoracic artery-radial artery composite grafts as the technique of choice for myocardial revascularization in elderly patients: a prospective randomized evaluation. *J Thorac Cardiovasc Surg*. 2004;127(1):179-184. doi:10.1016/j.jtcvs.2003.08.004

9. Lemma M, Mangini A, Gelpi G, Innorta A, Spina A, Antona C. Is it better to use the radial artery as a composite graft? Clinical and angiographic results of aorto-coronary versus Y-graft. *Eur J Cardiothorac Surg*. 2004;26(1):110-117. doi:10.1016/j.ejcts.2004.03.020

**Table 4** - Stone Brook Scar Evaluation Scale^3^

| Scar category |  | No. of point |
| --- | --- | --- |
| Width | >2 mm | 0 |
|  | ≤2 mm | 1 |
| Height | Elevated or depressed in relation to surrounding skin | 0 |
|  | Flat | 1 |
| Colour | Darker than surrounding skin (red, purple, brown or black) | 0 |
|  | Same colour or lighter than surrounding skin | 1 |
| Hatch marks or suture marks | Present | 0 |
|  | Absent | 1 |
| Overall appearance | Poor | 0 |
|  | Good | 1 |

**Table 5 -** Rating intervals of handgrip strength

|  | Males | | Females | |
| --- | --- | --- | --- | --- |
| Rating | (lbs) | (kg) | (lbs) | (kg) |
| excellent | >141 | >64 | >84 | >38 |
| very good | 123–141 | 56–64 | 75–84 | 34–38 |
| above average | 114–122 | 52–55 | 66–74 | 30–33 |
| average | 105–113 | 48–51 | 57–65 | 26–29 |
| below average | 96–104 | 44–47 | 49–56 | 23–25 |
| poor | 88–95 | 40–43 | 44–48 | 20–22 |
| very poor | <88 | <40 | <44 | <20 |

**Table 6** - Grading of muscle strength (Oxford Scale)

| Grade 0 | No muscle movement |
| --- | --- |
| Grade 1 | Muscle movement without joint motion |
| Grade 2 | Moves with gravity eliminated |
| Grade 3 | Moves against gravity but not resistance |
| Grade 4 | Moves against gravity and light resistance |
| Grade 5 | Normal strength |

**Table** 7 - Leeds Assessment of Neuropathic Symptoms and Signs (LANSS)^4^

| A. PAIN QUESTIONNAIRE | |
| --- | --- |
| • | Think about how your pain has felt over the last week. |
| • | Please say whether any of the descriptions match your pain exactly. |
| 1) | Does your pain feel like strange, unpleasant sensations in your skin? Words like pricking, tingling, and pins and needles might describe these sensations |
|  | a. NO – My pain doesn’t really feel like this……………………(0) |
|  | b. YES – I get these sensations quite a lot………………………(5) |
| 2) | Does your pain make the skin in the painful area look different from normal? Words like mottled or looking more red or pink might describe the appearance. |
|  | a. NO – My pain doesn’t affect the colour of my skin………….(0) |
|  | b. YES – I’ve noticed that the pain does make my skin look different from normal…………………………………………………………..(5) |
| 3) | Does your pain make the affected skin abnormally sensitive to touch? Getting unpleasant sensations when lightly stroking the skin, or getting pain when wearing tight clothes might describe the abnormal sensitivity. |
|  | a. NO – My pain doesn’t make my skin abnormally sensitive in that area….(0) |
|  | b. YES – My skin seems abnormally sensitive to touch in that area…………(3) |
| 4) | Does your pain come on suddenly and in bursts for no apparent reason when you’re still? Words like electric shocks, jumping and bursting describe these sensations. |
|  | a. NO – My pain doesn’t really feel like this…………….………(0) |
|  | b. YES – I get these sensations quite a lot……………………….(2) |
| 5) | Does your pain feel as if the skin temperature in the painful area has changed abnormally? Words like hot and burning describe these sensations. |
|  | a. NO – I don’t really get these sensations………………………(0) |
|  | b. YES – I get these sensations quite a lot……………………….(1) |
| B. SENSORY TESTING | |
| Skin sensitivity can be examined by comparing the painful area with a contralateral or adjacent non-painful area for the presence of allodynia and an altered pin-prick threshold (PPT). | |
| 1) | ALLODYNIA |
|  | Examine the response to lightly stroking cotton wool across the non-painful area and then the painful area. If normal sensations are experienced in the non-painful site, but pain or unpleasant sensations (tingling, nausea) are experienced in the painful area when stroking, allodynia is present. |
|  | a. NO, normal sensation in both areas……………………………(0) |
|  | b. YES, allodynia in painful area only……………………………(5) |
| 2) | ALTERED PIN-PRICK THRESHOLD |
|  | Determine the pin-prick treshold by comparing the response to a 23 gauge (blue) needle mounted inside a 2 ml syringe barrel placed gently on to the skin in the non-painful and then in the painful areas. |
|  | If a sharp pin-prick is felt in the non-painful area, but a different sensation is experienced in the painful area, e.g., none/blunt only (raised PPT) or a very painful sensation (lowered PPT), an altered PPT is present. |
|  | a. NO, equal sensation in both areas………………………………(0) |
|  | b. YES, altered PPT in painful area……………………………….(3) |
| SCORING: | |
| Add values in parentheses for sensory description and examination findings to obtain overall score. | |
| TOTAL SCORE (maximum 24) ……………. | |
| If score <12, neuropathic mechanisms are unlikely to be contributing to the patient’s pain. | |
| If score ≥12, neuropathic mechanisms are likely to be contributing to the patient’s pain. | |

**Figure 2 – Spatial definition of clinical neurological examination**


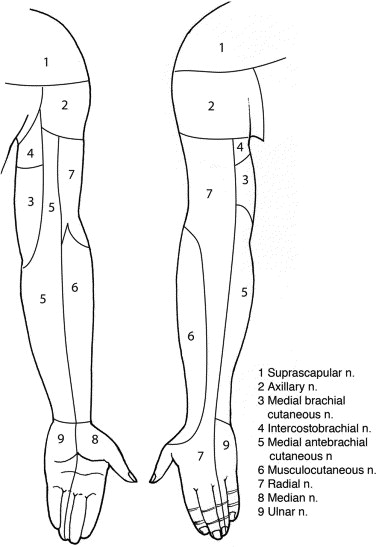


REFERENCES

1. Singleton JR, Bixby B, Russell JW, et al. The Utah Early Neuropathy Scale: a sensitive clinical scale for early sensory predominant neuropathy. *J Peripher Nerv Syst*. 2008;13(3):218-227. doi:10.1111/j.1529-8027.2008.00180.x

2. Zhu YY, Hayward PAR, Hadinata IE, et al. Long-term impact of radial artery harvest on forearm function and symptoms: A comparison with leg vein. *J Thorac Cardiovasc Surg*. 2013;145(2):412-419. doi:10.1016/j.jtcvs.2012.01.052

3. Singer AJ, Arora B, Dagum A, Valentine S, Hollander JE. Development and validation of a novel scar evaluation scale. *Plast Reconstr Surg*. 2007;120(7):1892-1897. doi:10.1097/01.prs.0000287275.15511.10

4. Bennett M. The LANSS Pain Scale: the Leeds assessment of neuropathic symptoms and signs. *Pain*. 2001;92(1-2):147-157.
